# Evidence-based interventions implemented in low-and middle-income countries for sickle cell disease management: A systematic review of randomized controlled trials

**DOI:** 10.1371/journal.pone.0246700

**Published:** 2021-02-17

**Authors:** Joyce Gyamfi, Temitope Ojo, Sabrina Epou, Amy Cohen, Lotanna Dike, Deborah Adenikinju, Scholastica Enechukwu, Dorice Vieira, Obiageli Nnodu, Gbenga Ogedegbe, Emmanuel Peprah

**Affiliations:** 1 Global Health Program, New York University School of Global Public Health, New York, New York, United States of America; 2 Department of Social and Behavioral Sciences, New York University School of Global Public Health, New York, New York, United States of America; 3 New York University Health Sciences Library, New York, New York, United States of America; 4 Centre of Excellence for Sickle Cell Disease Research & Training (CESRTA), University of Abuja, Abuja, Nigeria; 5 Department of Population Health, New York University Medical Center, New York, New York, United States of America; Qatar University, QATAR

## Abstract

**Background:**

Despite ~90% of sickle cell disease (SCD) occurring in low-and middle-income countries (LMICs), the vast majority of people are not receiving evidence-based interventions (EBIs) to reduce SCD-related adverse outcomes and mortality, and data on implementation research outcomes (IROs) and SCD is limited. This study aims to synthesize available data on EBIs for SCD and assess IROs.

**Methods:**

We conducted a systematic review of RCTs reporting on EBIs for SCD management implemented in LMICs. We identified articles from PubMed/Medline, Global Health, PubMed Central, Embase, Web of Science medical subject heading (MeSH and Emtree) and keywords, published from inception through February 23, 2020, and conducted an updated search through December 24, 2020. We provide intervention characteristics for each study, EBI impact on SCD, and evidence of reporting on IROs.

**Main results:**

29 RCTs were analyzed. EBIs identified included disease modifying agents, supportive care agents/analgesics, anti-malarials, systemic treatments, patient/ provider education, and nutritional supplements. Studies using disease modifying agents, nutritional supplements, and anti-malarials reported improvements in pain crisis, hospitalization, children’s growth and reduction in severity and prevalence of malaria. Two studies reported on the sustainability of supplementary arginine, citrulline, and daily chloroquine and hydroxyurea for SCD patients. Only 13 studies (44.8%) provided descriptions that captured at least three of the eight IROs. There was limited reporting of acceptability, feasibility, fidelity, cost and sustainability.

**Conclusion:**

EBIs are effective for SCD management in LMICs; however, measurement of IROs is scarce. Future research should focus on penetration of EBIs to inform evidence-based practice and sustainability in the context of LMICs.

**Clinical trial registration:**

This review is registered in PROSPERO #CRD42020167289.

## Introduction

Sickle cell disease (SCD), a progressively debilitating and chronic multi-organ genetic blood disorder characterized by anemia, severe pain and other vasoocclusive complications, and early mortality significantly impacts populations in low-and middle-income countries (LMICs). Globally, over 300,000 individuals are affected with SCD, with an estimated 90% occurring in LMICs [1]. Findings from a recent systematic review assessing SCD incidence and mortality globally suggest that Sub-Saharan Africa (SSA) bears the greatest mortality burden [1]. SCD contributes to 30–50% incidence of disability and unemployment, and is the leading cause of stroke in children and adolescents [2]. Comprehensive clinical care programs have reduced premature childhood deaths related to SCD by 70% in the United States of America (USA) [3]. In sharp contrast, it is estimated that 50–90% of children with SCD born in SSA die before the age of 5 [4, 5].

Despite the documented burden of SCD and proven therapies [1, 6, 7], the vast majority of people in LMICs are not receiving evidence-based health care (e.g., newborn screening, health education, prophylaxis for infection, optimal nutrition and hydration, blood transfusions, transcranial Doppler (TCD) screening, and hydroxyurea therapy), to reduce SC related adverse effects (i.e., reductions in pain crisis, acute chest syndrome, and hospitalizations) and mortality [7–10]. Effectiveness studies have documented patient, provider, and system level barriers including unavailability and unaffordable therapies and associated laboratory monitoring. Further, lack of provider knowledge and training, patient health belief, cost of therapy, patients or caregivers fears of side effects, providers’ uncertainty about safety of existing therapies, and regional differences in treatment guidelines are significant barriers to the use of evidence-based intervention (EBI). EBI is defined as treatments or interventions that have been shown to be effective through outcome evaluations such as randomized controlled trials (RCTs) or other rigorous methods, to address SCD in LMICs [11–18].

Our group has advocated for the utilization of implementation research to evaluate the delivery of these EBI for SCD in LMICs. Implementation research outcomes are “the effects of deliberate and purposive actions to implement new treatments, practices, and services” and have been described by Proctor and colleagues to include; *acceptability*, *adoption*, *appropriateness*, *costs*, *feasibility*, *fidelity*, *penetration*, *and sustainability* [19, 20]. Implementation research outcomes, outline how methods to promote the systematic uptake of research findings and other evidence-based practice into routine practice are assessed [21], and has the potential to identify the factors, processes, and methods that can successfully embed EBIs for SCD into policy and clinical practice [22]. Implementation outcome measures [20] are not well defined in the existing literature for SCD interventions in low-resource settings. Moreover, within the field of SCD research, an implementation research lens has yet to be applied to the delivery and evaluation of these interventions in LMICs in comparison to HICs [23, 24].

The objective of this study was to synthesize data for available EBI therapies implemented in LMICs for SCD management and identify whether implementation research outcomes were reported in published RCTs. A comprehensive understanding of uptake of effective SCD management therapies and resultant patient and implementation research outcomes will inform the complexities in treatment within underserved and vulnerable populations in LMICs. Evidence from this review will highlight the degree to which EBIs for SCD management are effective at reducing adverse outcomes in LMIC context, and the use and monitoring of implementation outcomes (e.g. adoption, etc.) to inform evidence-based practice in these settings.

## Methods

The review protocol is published in PROSPERO–(#CRD42020167289), Available from: https://www.crd.york.ac.uk/prospero/display_record.php?ID=CRD42020167289

### Search strategy

We developed a comprehensive search strategy to identify published trials that met predefined inclusion criteria using the standard Cochrane Collaboration systematic review technique [25] and the Preferred Reporting Items for Systematic reviews and Meta-Analysis (PRISMA) [26] and the World Bank criteria [27] were used to define LMICs. The following databases were searched: PubMed/Medline, Global Health, PubMed Central, Embase, Web of Science, Scientific Electronic Library Online (SCIELO), UNdata Online Library, Wiley Cochrane Library, and World DataBank. We searched grey literature in Google Scholar, ResearchGate, the New York Academy of Medicine (New York AM) Grey Literature database, and references of recently published systematic reviews on integrated chronic disease care. The article search was conducted on February 23, 2020 and was updated December 24, 2020. The full search strategy is provided in Appendix A (S1 File).

### Inclusion and exclusion criteria

Studies were included if they met the following inclusion criteria: 1) were published RCTs implemented in LMICs, 2) reported on various EBIs therapies for managing SCD within LMICs, and 3) were published in English. No limitation was placed on publication year and non-randomized studies, protocols, commentaries, and reviews (systematic, narrative, scoping, etc.) were excluded.

### Data extraction

All citations were downloaded to Covidence for title and abstract screening. Titles and abstracts of all articles were independently screened and rated by at least two reviewers to determine if they met the inclusion criteria. Discrepancies regarding eligibility of studies were resolved by discussion between raters. We then conducted full-text article review and extracted relevant information. Specifically, the following study characteristics were retrieved and coded: intervention type, duration, intervention setting, country, sample size, EBI therapies (e.g., *disease modifying agents*, *supportive care agents analgesics*, *antibiotics*, *pertinent vaccines systemic treatments*, *iron chelators*, *patient/carer/population education and nutritional supplements including folate supplementation*) recommended by the USA National Heart, Lung, Blood Institute [28] and World Health Organization (WHO) SCD management guidelines [29, 30] and statements inferring each implementation outcome as recommended by Proctor [20] were retrieved. We applied the definition of each implementation outcome to identify relevant information from the eligible articles. Reviewers used a standardized Google Form to extract relevant study data to address the research questions. Discrepancies were resolved by consensus or by additional reviewers. Data were imported to Excel and analyzed with SPSS statistical software version 27.

### Quality assessment

Risk of bias and quality of studies were assessed using the Cochrane Handbook for Systematic Review of Interventions, Version 5.1.0 [25] and the Cochrane risk-of-bias tool. Biases assessed included random sequence generation (selection bias), allocation concealment (selection bias), blinding of participants and personnel (performance bias), blinding of outcome assessment (detection bias), incomplete outcome data (attrition bias), and selective reporting (reporting bias). Quality of all trials was categorized as low/high/unclear risk of bias for each item mentioned above individually. Low risk of bias indicated that the item was well described and accounted for in the study; high risk of bias indicated the item was not sufficiently described in the study; and unclear risk of bias indicated that there was no information provided in the article to enable determination of the specific item of bias. All data were analyzed in Review Manager (RevMan 5.3).

## Results

**Fig 1:**
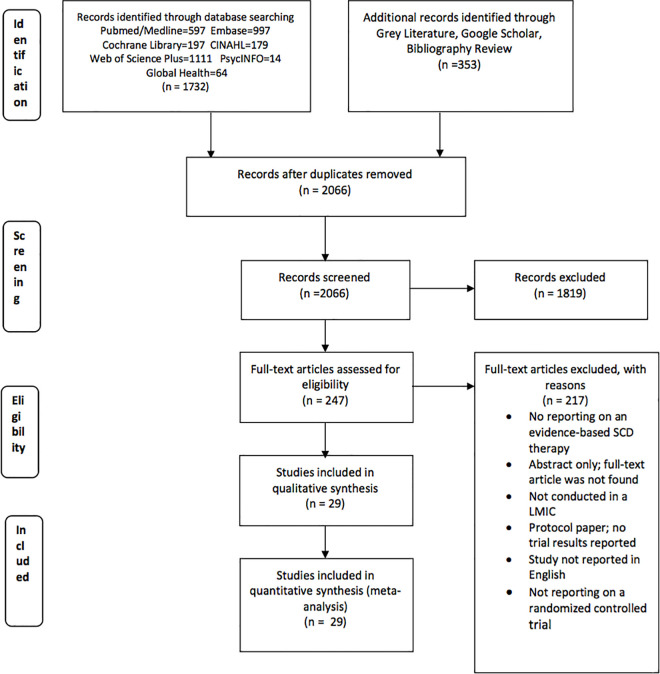
PRISMA flow diagram.

We retrieved 2085 articles and screened 2066 articles, after removing duplicates. Full-text review was conducted for 247 articles that initially met the inclusion criteria: study location in LMICs, reported on a therapy for SCD management, and published in English (Fig 1). After further review, we excluded 217 articles for the following reasons: articles reported on non- randomized controlled trial studies, studies that were not conducted in a LMIC, studies that did not report on an evidence-based SCD intervention, articles reporting on study protocols without reporting any trial results, articles reporting on systematic reviews, studies not published in English, and articles reporting only study abstracts whose full-text versions could not be located. Only 29 RCTs were included in this systematic review. The characteristics of the studies included are provided in Table 1.

**Table 1:** Characteristics of studies included in the systematic review.

Author (year)	Country	Study duration (months)	Sample Size	Female n (%)	Male n (%)	Evidence -Based Therapy	Target Population	Numbers in intervention and control groups	Completed follow-up at end of study	Completed follow-up at end of study	Primary outcome measure	Attainment of primary outcome
							(Adults/ Pediatric)	Intervention/Control	Intervention (%)	Control/comparison group (%)		YES / NO
Adjei et al. (2014) [33]	Ghana	1.4	119	nr	nr	Anti-malarial treatment artesunate-amodiaquine (AA), or artemether-lumefantrine (AL) (c)	Children	59/60	56 (94.9)	56 (93.3)	Parasite reduction ratios (PRR)	Yes (p-value or 95% CI not provided)
Alebouyeh et al. (2004) [34]	Iran	87.6	45	25 (55)	20 (44)	Hydroxyurea (a)	Children & Adult	36/9	nr	nr	Response to HU via increase in Hemoglobin levels beyond 10.0g/dl +-0.5	Yes (p-value or 95% CI not provided)
Arruda et al. (2012) [35]	Brazil	6	83	53 (64)	30 (36)	Vitamins C and E supplements	Adult	44/39	nr	nr	Reduction in haemolytic markers and acute complications	No (p>0.05)
Cox et al. (2018) [36]	Tanzania	18	145	nr	nr	Supplementary arginine, citrulline, and daily chloroquine	Children	61/58	58 (95%)	57 (98.3%)	Mean height-for-age Z-score and body-mass index-for-age Z-score	Yes (p = 0.001 for BMI-to-age Z-score; p = 0.081 for height-for-age Z-score)
											Mean differences between the RUSF-b and RUSF-v treatment groups in mean plasma amino acid concentrations (arginine, arginine to ornithine ratio, and arginine to ADMA ratio) and in FMDmax%.	
Daak et al. (2013) [37]	Sudan	12	140	61 (43)	79 (56)	Omega 3 capsules (a)	Children & Adult	67/61	58 (86.5)	55 (90)	Rates of clinical vaso-occlusive crisis and hemolytic events, blood transfusion rate, school attendance, and blood count	Yes (P-value<0.05)
Dawam et al. (2016) [38]	Nigeria	3	154	75 (48)	79 (51)	Sulphadoxine-Pyrimethamine, Proguanil (c, e)**	Children & Adult	77/77	72 (93.5)	70 (90.9)	Proportion of patients with malaria parasite in the peripheral blood	Yes (p-value = 0.01)
Dhabangi et al. (2015) [39]	Uganda	28	290	138 (47.5)	152 (52)	Red Blood Cell Transfusion (d)	Children	145/145	142/145 (97.9)	140/145 (96.6)	Difference in mean blood lactate level of 3mmol/L; Proportion of patients with a lactate level of 3 mmol/L or lower at 8 hours using a margin of noninferiority equal to an absolute difference of 25%	No (Hazard ratio = 0.99; 95% CI, 0.77 to 1.26)
Dhabangi et al. (2017) [40]	Uganda	20	147	66 (45)	81 (55)	Red Blood Cell Transfusion (d)	Children	70/77	nr	nr	B-type natriuretic peptide (BNP), vital signs, renal function tests, and plasma hemoglobin	No (p-value = 0.76)
Diop et al. (2011) [41]	Senegal	17	60	31 (51)	29 (48)	Sulfadoxine-pyrimethamine (c)	Adult	30/30	29 (96.7)	28 (93.3)	Prevalence of malaria	Yes (p-value or 95%CI not available)
Eke et al. (2003) [42]	Nigeria	9	101	51 (50.5)	50 (49.5)	Pyrimethamine and proguanil (c)	Children	71/30	Pyrimethamine: 36 (100) Proguanil: 32 (91.4)	Placebo: 29 (96.7)	Clinical and laboratory features of malaria (presence of parasitemia, parasite count and density, hepatomegaly and/or splenomegaly, symptomatic malarial infection [42], bone pain crises, and hemolytic crises)	Yes (parasite density: p-value = 0.045)
Eleuterio et al. (2019) [43]	Brazil	4	50	26 (52)	24 (48)	L-arginine (500 mg) + hydroxyurea (a)**	Adult	25/25	nr	nr	Nitrite and pain levels	Yes (p<0.001)
Farber, M et al. (1991) [44]	Jamaica	84	116	55 (47.4)	61 (52.5)	Photocoagulation Therapy (a)	Adult	99/75	nr	nr	Reduction of Proliferative Sickle Cell Retinopathy (PSR) through reduced visual acuity loss	Yes (p = 0.019)
Galadanci et al. (2017) [45]	Nigeria	3	235	122 (52)	113 (48)	Hydroxyurea (a)	Children	25/210	23 (96.0)	196 (93.3)	Recruitment, retention, and adherence rates to hydroxyurea therapy	Yes (p-value not reported)
Hankins et al. (2015) [32]	Jamaica, US, Brazil	30	22	14 (64)	8 (36)	Hydroxyurea (a)	Children	11/11	nr	nr	Cumulative incidence of conversion to abnormal maximum TAMV velocities (in any of the 10 vessels measured)	Yes (p = 0.02)
Heeney et al. (2016) [31]	SSA- Ghana, Kenya	24	341	173 (50.7)	163 (49)	Prasugrel (a)	Children	171/170	171 (100)	170 (100)	Between group difference in reduced rate of vaso-occlusive crisis, a composite of painful crisis or acute chest syndrome	No (p = 0.12)
	EU- Belgium, UK, Italy											
	Americas- Canada, US, Brazil											
	North Africa/Middle East- Saudi Arabia, Egypt, Oman, Lebanon, Turkey											
Inusa et al. 2019 [17]	SSA- Ghana, Kenya	24	341	173 (50.7)	168 (49)	Prasugrel (a)	Children	171/170	171 (100)	170 (100)	Rate of vaso-occlusive crisis by region, a composite of painful crisis or acute chest syndrome	Yes (p = 0.003)
	EU- Belgium, UK, Italy											
	Americas- Canada, US, Brazil											
	North Africa/Middle East- Saudi Arabia, Egypt, Oman, Lebanon, Turkey											
Jain et al. (2012) [15]	India	18	60	32 (53)	28 (47)	Hydroxyurea (a)	Children	30/30	30 (100)	30 (100)	Frequency of vasoocclusive crises per patient per year	Yes (p<0.001)
Kutlar et al. (2013) [46]	Lebanon, Egypt, Jamaica	6	52	24 (46)	28 (54)	Dimethyl butyrate (a)	Children	21/31	nr	nr	Effects of HQK-1001 on Hb F	Yes (p value and 95% CI not available)
Kutlar et al. (2018) [47]	Brazil, Jamaica, US	12	198	171 (86)	27 (14)	Crizanlizumab (a)	Children	67/65	63 (94)	59 (90.7)	Proportion of patients that are VOC-free between crizanlizumab vs placebo groups	Yes (p value and 95% CI not available)
			US (151) patients), Brazil (40), and Jamaica(7)									
La Grenade et al. (1993) [48]	Jamaica	6	32	14 (44)	18 (56)	Solcoseryl & duoderm (d)	Adult	24/20	nr	nr	Increase in ulcer healing; reduced ulcer size	Yes but non-significant change (p>0.05)
Manrique et al. (1987) [49]	Brazil	1.4	60	23 (38)	37 (62)	Pentoxifylline (b)	Children & Adult	30/30	nr	nr	Pain frequency, intensity, and duration	Yes (p<0.05)
Martins et al. (2009) [50]	Brazil	3	60	38 (63)	22 (37)	Alpha-lipoic acid (antioxidant)	Children & Adult	60	nr	nr	Prevention of oxidative damage	Yes (p<0.05)
Misra et al. (2017) [16]	Panama, Colombia	0.25	24	15 (63)	9 (38)	Sanguinate (pegylated bovine carboxyhemoglobin), hydroxyurea (a,b) **	Adult	12/12	nr	nr	Safety of Sanguinate	Yes (p-value or 95%CI not given)
Nakibuuka et al. (2009) [51]	Uganda	5	242	121 (50)	121 (50)	Chloroquine, sulphadoxine-pyrimethamine (SP) (c)	Children	120/122	113 (94.1)	114 (93.4)	Reduced prevalence of malaria episodes	Yes (p = 0.042)
Olaosebikan et al. (2015) [52]	Nigeria	19	270	129 (48)	141 (52)	Intermittent preventive treatment (IPT) with a fixed-dose combination of mefloquine-artesunate (MQAS) or sulfadoxine-pyrimethamine plus amodiaquine (SPAQ) (c)	Children & Adult	180/90	MQAS 69 (76.7) SPAQ 75 (83.3)	Proguanil 60 (66.7)	Occurrence of any adverse event measured in rate ratio	Yes
												Rate ratio, 95% CI: MQAS = 0.68 (0.51–0.91); SPAQ = 0.74 (0.55–0.98)
Olupot-Olupot et al. (2014) [53]	Uganda	4	160	81 (51)	79 (49)	Blood Transfusions (d)	Children	78/82	71 (91)	70 (85.3)	Correction of severe anemia (to hemoglobin >6 g/dl) at 24 hours	Yes (p = 0.01)
Opoka et al. (2017) [54]	Uganda	12	208	96 (46)	112 (54)	Hydroxyurea (a)	Children	104/103	nr	nr	Incidence of clinical malaria	No (p = 0.61)
Uke et al. (2000)	Nigeria	9	58	21 (36)	37 (64)	Oral piroxicam with soluble aspirin (b)	Children	29/29	29 (100)	29 (100)	Pain relief, limitation of movement, fever, and insomnia or agitation	Yes (p-value<0.05)
Wambebe et al. (2001)#1 [55]	Nigeria	12	82	38 (55)	31 (45)	Niprisan (a)	Children & Adult	33/36	33 (100)	36 (100)	Reduction in the frequency of crises, severe pain, absenteeism from work, and hospitalizations	Yes (p<0.05)
Wambebe et al. (2001)#2 [56]	Nigeria	12	82	46 (56)	36 (44)	Niprisan (a)	Children & Adult	34/36	34 (100)	36 (100)	Reduce vaso-occlusive crisis	Yes (p<0.01)

• The sample size reported is the total number of patients (HICs + LMICs) and not just LMICs for studies that included non-LMICs

• nr: not reported

• Disease Modifying Agents (15 studies); (b) Supportive Care Agents Analgesics (3 studies); (c) Anti-Malarials (6 studies); (d) Systemic Treatments (4 studies); (e) Patient/Carer/Population Education (1 study); (f) Nutritional Supplements (3 studies)

** Entries with the symbol indicate the use of interventions in more than one category (n = 3).

The review included 30 papers that reported on 29 RCTs conducted in 14 LMICs. Heeney et al. [31] and Inusa et al. [17] were two papers reporting on the same study. Seven studies were conducted each in Nigeria and Brazil, 5 studies each in Jamaica and Uganda, 2 studies each in Ghana, Egypt and Lebanon, and 1 study each in Iran, Kenya, Sudan, Senegal, Turkey, Colombia, Tanzania and India. Five studies were conducted at multiple sites; Hankins et al. [32] and Kutlar et al. [47] conducted studies in Jamaica, US, and Brazil; Inusa et al. [17] and Heeney et al. [31] conducted studies in Ghana, Kenya, Belgium, UK, Italy, Canada, US, Brazil, Saudi Arabia, Egypt, Oman, Lebanon, Turkey; Kutlar et al. [46] conducted studies in Lebanon, Egypt, Jamaica; Misra et al. [16] conducted studies in Panama and Colombia. Of these LMICs, 18 studies were conducted in Africa—Nigeria (n = 7), Uganda (n = 5), Egypt (n = 1), Tanzania (n = 1), Ghana (n = 1), Sudan (n = 1), Senegal (n = 1), Kenya (n = 1); 8 studies were conducted in Latin America—Brazil (n = 7) and Colombia (n = 1); 5 were conducted in the Caribbean–Jamaica (n = 5); 2 were conducted in the Middle East—Iran (n = 1) and Lebanon (n = 1) and 2 studies were conducted in Europe and Asia—India (n = 1) and Turkey (n = 1).

Majority of the studies were conducted in a hospital/clinic setting, with only one study conducted in a University setting. Studies lasted between 7 days and 87.6 months with a mean study duration of 16.1 months (SD: 20.26 months); study participants ranged from 32 to 341 participants with a mean sample size of 125 participants (SD: 94.11) and inclusion of 14 to 173 female participants, with a mean sample size of 65 (SD: 48.33).

Eight studies reported a combination of children and adults as the target population; 15 studies targeted children only and 6 studies solely targeted adults. Reported participant follow-up for 24 studies ranged from 2 days to 47.4 months [57], with most intervention completion rates being higher than 83% (n = 15); with exception of one study with the lowest completion rates for both intervention (76.7%) and control (66.7%) [52]. Fourteen studies did not report follow-up completion rates. Sixteen studies reported statistically significant difference between the intervention and control groups for the primary outcome with either a p-value <0.05 or a 95% CI outside of 0 (absolute value) or 1 (relative value) while 4 studies reported lack of desired primary intervention outcome with a p>0.05 or a 95% CI that includes 0 (absolute value) or 1 (relative value).

**Fig 2:**
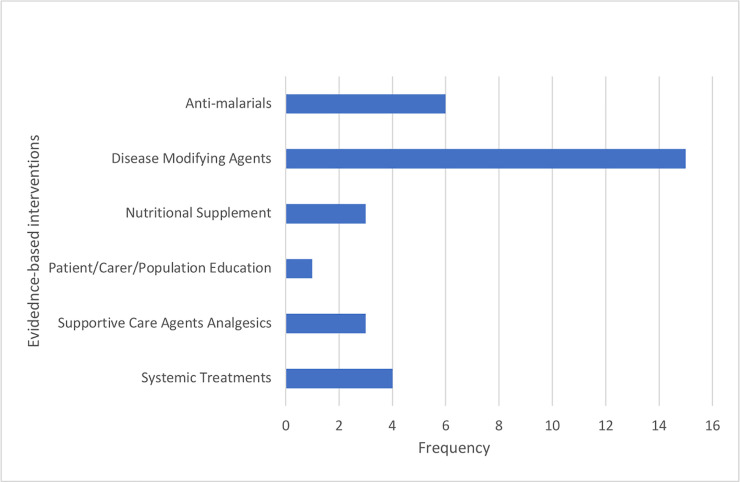
Classification of evidence-based interventions used in selected studies.

The studies utilized evidence based interventions in one of the following categories: Disease Modifying Agents (n = 15), Supportive Care Agents Analgesics (n = 3), Anti-Malarials (n = 6), Systemic Treatments (n = 4), Patient/Carer/Population Education (n = 1), and Nutritional Supplements (n = 3) (Table 1, Fig 2). Three studies utilized multiple evidence-based interventions.

All included studies were assessed on the following implementation outcomes measures: Adoption, Appropriateness, Acceptability, Cost, Feasibility, Fidelity, Penetration and Sustainability. Of the studies reviewed, 16 reported on adoption, 19 on appropriateness, 10 on acceptability, 7 on cost, 12 on feasibility, 8 on fidelity, and 2 on sustainability (Table 2). There was insufficient information to determine penetration (level of institutionalization /spread) from any of the studies. Thirteen (44.8%) of the 29 studies reported on 3 or more implementation outcomes.

**Table 2:** Implementation outcomes inferred from studies.

	Implementation outcomes and general definitions						
Author (year) / Country	Adoption	Appropriateness	Acceptability	Cost	Feasibility	Fidelity	Sustainability
	(uptake, utilization, intention to try)	(perceived fit, relevance, compatibility, suitability, usefulness, practicability)	(satisfaction with evidence-based intervention)	(cost, cost-effectiveness, cost-benefit)	(actual fit or utility, practicability for the population or setting)	(delivered as intended, adherence, integrity, quality of program delivery)	(maintenance or sustained use of the intervention post initial implementation)
Adjei et al. (2014) [33] Ghana	x	x	x		x		
Alebouyeh et al. (2004) [34] Iran	x	x	x		x		x
Arruda et al. (2012) [35] Brazil	x	x	x		x	x	
Cox et al. (2018) [36] Tanzania	x	x	x		x		x
Daak et al. (2013) [37] Sudan			x	x	x		
Dawam et al. (2016) [38] Nigeria	x			x			
Dhabangi et al. (2015) [39] Uganda		x				x	
Dhabangi et al. (2017) [40] Uganda							
Diop et al. (2011) [41] Senegal			x	x		x	
Eke et al. (2003) [42] Nigeria	x	x		x		x	
Eleuterio et al. (2019) [43] Brazil	x						
Farber, M. (1991) [44] Jamaica	x						
Galadanci et al. (2017) [45] Nigeria	x		x	x	x		
Hankins et al. (2015) [32] Jamaica, US, Brazil	x	x					
Heeney et al. (2016) [31] Kenya		x					
Inusa et al. (2019) [17] Kenya		x				x	
Jain et al. (2012) [15] India	x	x					
Kutlar et al. (2013) [46] Lebanon, Egypt, Jamaica		x				x	
Kutlar et al. (2018) [47] Brazil, Jamaica, US		x	x				
La Grenade et al. (1993) [48] Jamaica	x	x	x				
Manrique et al. (1987) [49] Brazil	x	x				x	
Martin et al. (2009) [50] Brazil		x	x				
Misra et al. (2017) [16] Colombia					x		
Nakibuuka et al. (2009) [51] Uganda		x			x	x	
Olaosebikan et al. (2015) [52] Nigeria	x	x		x	x		
Olupot-Olupot et al. (2014) [53] Uganda	x	x		x	x		
Opoka et al. (2017) [54] Uganda	x	x					
Uke et al. (2000) [58] Nigeria					x		
*Wambebe (2001)#1 [55] Nigeria					x		
*Wambebe (2001) #2 [56] Nigeria							

* Wambebe (2001)#1 [55] assessed the efficacy and tolerability of niprisan in the management of patients with sickle cell disease, this is a secondary study of the main clinical trial reported in Wambebe (2001)#2 [56].

Adoption was reported as optimal adherence/compliance to the trial protocol by both providers and patients [35, 36, 38], participants showing up for most or all of the scheduled intervention visits [33, 34, 44, 45], or successful enrollment into the study [53]. Likely non-adoption of an intervention was reported as the difficulty of patients to comply with daily or weekly use of medications (prophylaxis) [52, 54] or slow patient accrual and administrative delays [32].

Appropriateness, understood as perceived fit of an intervention can be inferred from trials that attained desired outcomes, specifically outcomes assessing safety and effectiveness of such intervention [39, 51]. Inappropriate interventions are reported as lack of evidence to support the desired intervention outcome of optimal survival of patients [53] or not recommended for the study participants [35].

Acceptability was mainly reported from the participants’ perception of the intervention. Acceptable interventions were reported as being well tolerated by SCD patients [48] or participants reporting a better quality of life [41]; unacceptable interventions were reported as not well tolerated by patients or as interventions from which patients’ experienced adverse outcomes or side effects [47, 48]or not recommended for use for the target population [59].

Interventions were reported as feasible in summary statements that suggest interventions should be further replicated [37], confirmation of desired intervention outcome [34, 35, 60] that interventions were ‘feasible and safe’ to implement [44, 45, 53]. Non-feasible interventions were those that exhibited side effects that might deter recommendation or use of the intervention [35].

Implementation costs were reported as comparative average cost of purchasing SCD medications [38, 52] or existing out-of-pocket expenses associated with accessing the intervention [45]. Other studies reported an SCD intervention as either ‘affordable’[37] or with substantial cost saving potential, but with need for further research to assess its cost-effectiveness [53].

Fidelity was reported as adherence/compliance to intervention activities by study participants [35, 46] or study implementers/administrators [39, 51]. Some tools used to assess fidelity of intervention included diaries of pill counts and self-assessment sessions during follow-up visits [49].

Sustainability of evidence-based SCD interventions was reported through factors that could influence sustainability, such as side-effects of long term use of a SCD medication (e.g. hydroxyurea, side effect on children’s growth and development) [34] or increase in pain-related adverse events, in spite of a safe and well-tolerated medication like RUSF [36] or the potential that an intervention will lose its effect over time, given its small effect size in the present study [36].

### Quality of studies

**Fig 3:**
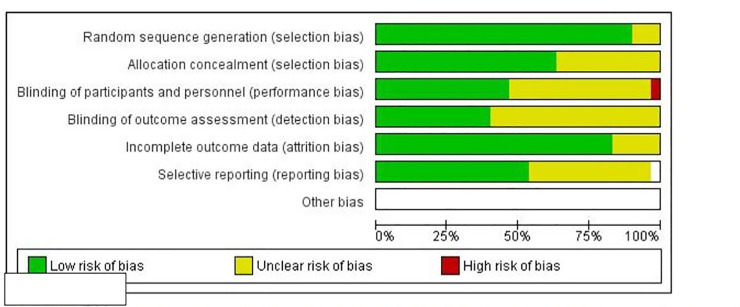
Risk of bias graph: Review authors’ judgements about each risk of bias item presented as percentages across all included studies.

The Cochrane Risk of Bias tool was used to assess the 29 RCTs included in this review. Random sequence generation and allocation concealment together represent selection bias. More than 75% of the studies had a low risk of selection bias due to random sequence generation [15–17, 31–44, 46–48, 51–56, 58]; about 62.5% of the studies also had a low risk of selection bias due to allocation concealment [15, 17, 33, 35–42, 47, 51–54, 56, 61]. Less than 50% of studies had a low risk of performance bias due to blinding of participants and personnel [15, 32, 35–39, 42, 43, 47, 51–54, 56]; about 40% of studies had a low risk of detection bias due to blinding of outcome assessment [32, 33, 36, 39, 40, 47, 51–53, 56, 61, 62]; more than 75% of the studies had a low risk of attrition bias due to incomplete outcome data [16, 32–42, 44–47, 49, 51–56, 61, 62];and over 50% of the studies had a low risk of reporting bias due to selective reporting [32, 33, 40, 44–47, 49, 51–56, 63] (Fig 3 and S1 Table).

## Discussion

We identified the various recommended EBIs including hydroxyurea, anti-malarials, transfusion and alternative management [7, 64] implemented in LMICs for SCD management and evaluated implementation outcomes inferred by statements provided by the authors. Effective medications such as hydroxyurea–the only approved drug therapy by the USA Food and Drug Administration which modifies the disease pathogenesis and increases fetal hemoglobin, has “… transformed SCD from a life-threatening disease for children to chronic diseases for adults” [65]. Hydroxyurea is the disease modifying drug of choice used by most studies reviewed (Table 1). However, it is important to acknowledge other alternative methods that SCD patients use to manage pain crisis within LMICs. These may include traditional herbal medications and prayer [66, 67]. Also, patients’ perception of medication(s) should be considered including fear of side effects from western medication and limited knowledge of potential risk and benefits of the therapy. Patients in these settings often lack informed knowledge about therapies [68] and therefore are more likely to be noncompliant.

Moreover, although EBIs are cost effective because of the resultant reduction in hospital visits, admission rates, frequency of pain crisis and other SCD related morbidities which makes up for the relative expense of the EBIs, it may pose undue economic burden to patients and their families in low resource settings. For example, since hydroxyurea is taken orally, once a day in the form of a pill, obtaining sufficient amount of medication coupled with the cost associated with regular laboratory monitoring may be challenging and thus the medication is likely to be underutilized in low-resource settings [69–71]. Furthermore, blood transfusions may be unsafe, costly, and unaffordable in LMICs. Also, although gene therapy may show promise of alleviating SCD in higher-income countries [72, 73], this therapy is inaccessible to individuals in LMICs.

Findings from this review illuminate the scant use of disease modifying agents, supportive care agents, analgesics, anti-malarials, systemic treatments, nutritional supplements, and patient/carer/population education in LMICs. Only one study imparted health education to patients and care givers. Moreover, there was limited reporting of implementation outcomes, specifically acceptability, feasibility, fidelity, cost and sustainability. Although implementation outcomes were not the primary outcomes of the studies reviewed, reporting pertinent information such as sustainability, as done for two evidence-based SCD therapies, namely hydroxyurea and supplementary arginine, citrulline, and daily chloroquine, is important to ensure that future SCD patients can benefit from these treatments.

Furthermore, it is important to acknowledge the unique challenge of limited resources such as unavailable and costly medications and laboratory monitoring relative to average income and the fact that health expenditure is out of pocket with no health insurance options in most LMICs compared to higher-income countries, when introducing new therapies for SCD care. EBIs targeted at SCD management must address patient, provider, and system-level barriers of care delivery, as findings from a recent Cochrane systematic review indicates that effective strategies for implementation of EBI in LMICs are those that involve a multi-level approach, and are tailored to the context of the built-environment [74]. Building capacity at multiple levels will improve EBI adoption for SCD management in LMICs. At the systems level, availability of therapy at a low cost will ensure that patients are able to afford the medication. At the provider-level, training physicians (including non-hematologist) and other healthcare providers (e.g., nurses) in the appropriate use and management of side effects of therapies may improve prescription practices [75, 76] and also ensure that some aspects of SCD management such as health maintenance can be carried out at the primary care level by educating the staff at that level via task strengthening. Moreover, SCD is a multisystemic disease requiring the involvement of many specialists in the management process. At the patient-level, involvement of caregivers, newborn screening and early intervention, provision of health education about therapeutic management for SCD, and knowledge on when families should seek referral for care may ensure timely care and improve patients condition [77].

Upon updating our search in December 2020, 62 additional articles were retrieved. Of the 62, seven were selected for full-text review based on the title and abstract screening [70, 78–83]. However, after thorough review, all of the studies were excluded for reasons including not conducted in LMICs, reviews, and or commentaries.

### Strengths and limitations

This study used a rigorous search strategy based on a pre-specified inclusion and exclusion criteria to retrieve articles across multiple databases. No restriction was placed on article publication date in order to capture all relevant articles. The limitations include the assessment of RCT articles published in English only. Evidence from non-English studies and studies using other design strategies (i.e., pre/post) may have been missed. However, non-RCT studies may not provide strong evidence to observe a sustained intervention effect of the EBIs used. Also, the heterogeneity in terms of the various therapies did not allow for a meta-analysis to be conducted.

## Conclusion

To reduce SCD related morbidity and mortality, the therapies discussed in this review should be implemented in synergy with ensuring the uptake and sustainability of resource intensive EBIs [84–86]. Future studies undertaken in LMICs that face suboptimal infrastructure and resources, must employ implementation research methodology whilst engaging key stakeholders (providers and patients) and integrate findings from implementation outcome assessment into evidence-based practice for SCD management.

## Supporting information

S1 File Appendix A: Search strategy. (PDF)

S2 File PRISMA 2009 checklist. (PDF)

S1 Table Risk of bias summary table for included studies. (DOCX)

## Abbreviations:

EBI: Evidence-Based Interventions
LMICs: Low and middle income countries
SCD: Sickle Cell Disease
SSA: Sub-Saharan Africa
TCD: Transcranial Doppler
IROs: Implementation Research Outcomes
