# Supplementary material for: Evidence-based interventions implemented in low-and middle-income countries for sickle cell disease management: A systematic review of randomized controlled trials
Source: PLoS One. 2021 Feb 17;16(2):e0246700. doi: 10.1371/journal.pone.0246700 (PMC7888630; doi:10.1371/journal.pone.0246700)
Supplement: S1 File — (PDF) [file pone.0246700.s001.pdf]

S1.File.Appendix A: Search Strategy

Evidence-based Interventions Implemented in Low-and Middle-Income Countries for Sickle Cell Disease Management: A systematic review of randomized controlled trials

Final Search strategy (PubMed)

(evidence-based interventions OR evidence based practice OR health care outcomes assessment OR clinical trials OR evidence based medicine OR comparative effectiveness OR Interventions OR clinical trials OR clinical trial OR outcome studies OR outcome measures OR outcomes assessment OR randomized controlled trial OR clinical study OR clinical studies OR randomized OR randomised OR implementation OR implementation science OR randomized control trial) **AND** (sickle cell disease OR sickle cell anemia OR acute chest syndrome OR hemoglobin SC disease OR HbS disease) **AND** (management OR therapy OR treatment OR therapies OR therapeutics OR pain Management) **AND** (developing countries OR developing country OR medically underserved area OR medically underserved areas lmic OR low income countries OR low income country OR middle income countries OR middle income country OR resource poor OR low resource OR third world country OR third world countries OR less developed country OR less developed countries OR least developed country OR least developed countries OR africa OR central asia OR western asia OR southeastern asia OR indian ocean islands OR central america OR south america OR eastern europe OR transcaucasia OR caribbean region OR pacific islands OR afghan OR afghan OR afghanistan OR bangladesh OR bangladeshi OR benin OR beninese OR burkina faso OR burkinabes OR burundi OR burundian OR cambodia OR cambodian OR central african republic OR central african OR chad OR chadian OR comoros OR comorian OR congo OR congolese OR eritrea OR eritrean OR ethiopia OR ethiopian OR gambia OR gambian OR guinea OR guinea OR haiti OR haitian OR kenya OR kenya OR korea OR korean OR kyrgyz OR kyrgyzstan OR liberia OR liberian OR madagascar OR malagasy OR malawi OR malawian OR mali OR malian OR mozambique OR mozambican OR myanmar OR myanmarese OR burmese OR nepal OR nepalese OR niger OR nigerian OR rwanda OR rwandan OR sierra leone OR sierra leoneans OR somalia OR somalia OR tajikistan OR tajik OR tadzhik OR tanzania OR tanzanian OR togo OR togolese OR uganda OR ugandan OR zimbabwe OR zimbabwean OR angola OR angola OR armenia OR armenian OR belize OR belizean OR bhutan OR bhutanese OR bolivia OR bolivian OR cameroon OR cameroonian OR cape verde OR cape verdeans OR cape verdeans OR cote d'ivoire OR ivory coast OR djibouti OR egypt OR egyptian OR el salvador OR salvadoran OR fiji OR fijian OR georgia OR georgia OR ghana OR ghanaian OR guatemala OR guatemalan OR guyana OR guyanese OR honduras OR honduran OR indonesia OR indonesian OR india OR indian OR iraq OR iraqi OR kiribati OR kosovo OR kosovar OR laos OR Lao OR laotian OR lesotho OR marshall islands OR marshallese OR mauritania OR mauritanian

OR micronesia OR micronesian OR moldova OR moldovan OR mongolia OR mongolian OR morocco OR moroccan OR nicaragua OR nicaraguan OR nigeria OR nigerian OR pakistan OR pakistani OR papua new guinea OR papua new guinean OR paraguay OR paraguayan OR philippines OR filipino OR samoa OR samoan OR sao tome OR principe OR santomea OR senegal OR senegalese OR solomon islands OR solomon islander OR sri lanka OR sri lankan OR sudan OR sudanese OR swazi OR swaziland OR syria OR syrian OR east timor OR east timor eye OR tonga OR tongan OR turkmenistan OR turkmen OR tuvalu OR tuvaluans OR ukraine OR ukrainian OR uzbekistan OR uzbek OR vanuatu OR vietnam OR vietnamese OR west bank OR gaza OR palestinian OR yemen OR yemeni OR yemenite OR zambia OR zambian OR albania OR albanian OR algeria OR algerian OR argentina OR argentinian OR azerbaijan OR azerbaijani OR belarus OR belarusian OR bosnia OR bosnian OR botswana OR brazil OR brazilian OR bulgaria OR bulgarian OR china OR chinese OR colombia OR colombian OR costa rica OR costa rican OR cuba OR cuban OR dominica OR dominican OR ecuador OR ecuadorean OR gabon OR gabonese OR grenada OR grenada OR iran OR iranian OR jamaica OR jamaican OR jordan OR jordanian OR kazakhstan OR kazakhstan OR lebanon OR lebanese OR libya OR libyan OR lithuania OR lithuanian OR macedonia OR macedonian OR malaysia OR malaysian OR maldives OR maldivian OR mauritius OR mauritian OR mexico OR mexican OR montenegro OR montenegrin OR namibia OR namibian OR palau OR palauan OR panama OR panamanian OR peru OR peruvian OR romania OR romanian OR russia OR russian OR serbia OR serbian OR seychelles OR seychellois OR south africa OR south african OR saint kitts OR saint lucia OR saint vincent OR surinam OR suriname OR suriname OR thailand OR thai OR tunisia OR tunisian OR turkey OR turkish OR venezuela OR venezuela OR venezuelan OR venezuelan OR herzegovina OR timor leste OR dominican republic OR grenadines OR american samoa OR american samoan OR guinea bissau OR bissau guinean)

Notes: The above search strategy was replicated for the other database search engines.

PubMed's Medical Subject Headings (MeSH) database and Embase's Emtree thesaurus were used to ensure that relevant subject headings were searched.
