# Supplementary material for: Evidence-based interventions implemented in low-and middle-income countries for sickle cell disease management: A systematic review of randomized controlled trials
Source: PLoS One. 2021 Feb 17;16(2):e0246700. doi: 10.1371/journal.pone.0246700 (PMC7888630; doi:10.1371/journal.pone.0246700)
Supplement: S1 Table — (DOCX) [file pone.0246700.s003.docx]

Uke 2000

Uke 2000

La GrenadeGrenade

/

**S1 Table. Risk of Bias Summary Table for included studies**
